# Antitumor Research on Artemisinin and Its Bioactive Derivatives

**DOI:** 10.1007/s13659-018-0162-1

**Published:** 2018-04-09

**Authors:** Yunqin Zhang, Guowei Xu, Shuqun Zhang, Dong Wang, P. Saravana Prabha, Zhili Zuo

**Affiliations:** 1 State Key Laboratory of Phytochemistry and Plant Resources in West China, Kunming Institute of Botany, Chinese Academy of Sciences, Kunming, 650201 China; 2 University of the Chinese Academy of Sciences, Beijing, 100049 China; 3 Yunnan Key Laboratory of Natural Medicinal Chemistry, Kunming, 650201 Yunnan China

**Keywords:** Artemisinin, Artemisinin derivatives, Antitumor, Activity, Mechanism

## Abstract

**Abstract:**

Cancer is the leading cause of human death which seriously threatens human life. The antimalarial drug artemisinin and its derivatives have been discovered with considerable anticancer properties. Simultaneously, a variety of target-selective artemisinin-related compounds with high efficiency have been discovered. Many researches indicated that artemisinin-related compounds have cytotoxic effects against a variety of cancer cells through pleiotropic effects, including inhibiting the proliferation of tumor cells, promoting apoptosis, inducing cell cycle arrest, disrupting cancer invasion and metastasis, preventing angiogenesis, mediating the tumor-related signaling pathways, and regulating tumor microenvironment. More importantly, artemisinins demonstrated minor side effects to normal cells and manifested the ability to overcome multidrug-resistance which is widely observed in cancer patients. Therefore, we concentrated on the new advances and development of artemisinin and its derivatives as potential antitumor agents in recent 5 years. It is our hope that this review could be helpful for further exploration of novel artemisinin-related antitumor agents.

**Graphical Abstract:**

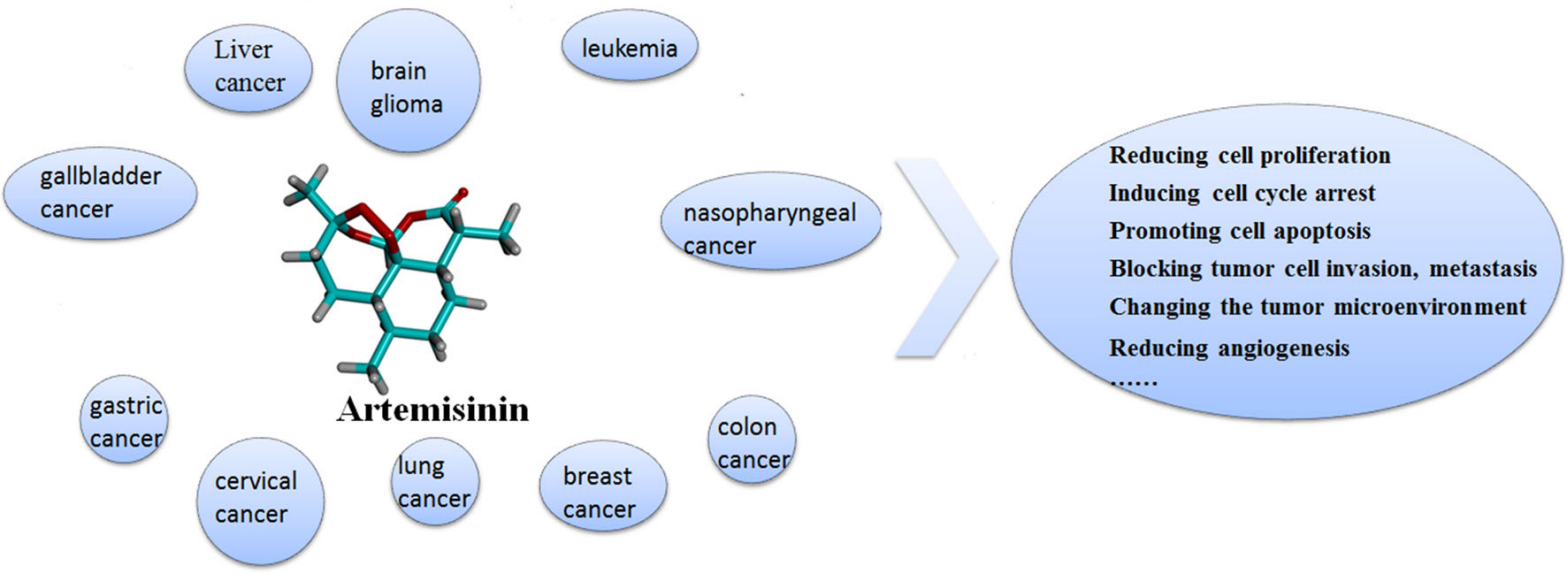

## Introduction

Cancer is currently a worldwide public health issue, which is, statistically, rated as the leading cause of death. Conventional cancer therapies consisting of chemotherapy, surgery, and radiotherapy may have limited efficacy, especially with the problem of drug resistance [1]. In spite of many significant progresses, cancer is still a major disease that causes more than 8 million deaths every year, or about 15% of all human deaths around the world. Moreover, the data from the WHO suggested that 22 million people could develop cancer annually within the next two decades, while cancer deaths are predicted to rise to 13 million per year [2]. However, with the current chemotherapeutic efficacy against carcinoma still unsatisfactory, the discovery of potent, safe, and selective antitumor drugs could cause a huge scientific and commercial interest from many academic researchers.

**Fig. 1:**
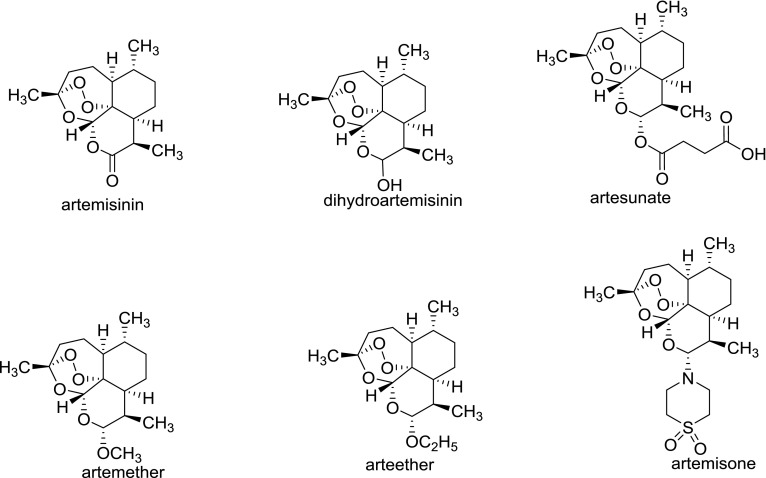
Chemical structures of artemisinin and its common bioactive derivatives

Traditional Chinese Medicine (TCM) and related natural active ingredients provide rich resources for the development of modern medicine. For instance, artemisinin (ART), a sesquiterpene lactone that bears a peroxide grouping, was isolated by Chinese pharmacist Youyou Tu et al. from the leaf of the herb *Artemisia annua* L. (sweet wormwood). As the first-line drug for the treatment of human malaria, artemisinin and its derivatives (Fig. 1) has been recognized as the most potent treatment for malaria in the world. With the further development of artemisinin and its derivatives, studies have found that artemisinins also have desirable antitumor activity in human cancer treatment. Additionally, derivatives of artemisinin, such as dihydroartemisinin (DHA), artemether (ATM), arteether, artemisone, and artesunate (AS), appear to be more potent than artemisinin. However, the mechanisms of action are incompletely elucidated. It appears that iron-mediated cleavage of the endoperoxide bridge plays a vital role in achieving their anti-cancer properties. Many researches have pointed out that cancer cells contain significantly more intracellular free iron than normal cells, while artemisinin contains an endoperoxide moiety can react with iron to form cytotoxic free radicals [3]. It has been shown that artemisinin and its analogs selectively cause apoptosis in multiple cancer cell lines [4–11]. In addition, artemisinin-related compounds have been shown to possess amount of antitumor related properties, such as suppressing the cells proliferation, inducing apoptotic response, arresting tumor cell cycle, inhibiting cells invasion and metastasis, preventing angiogenesis, altering oxidative damage reactions, disrupting cancer signaling pathway, and regulating tumor microenvironment [12–25]. These properties make artemisinin-related compounds becoming a series of attractive cancer chemotherapeutic drug candidates.

During the recent years, researches are gradually repositioning artemisinin and its analogs as promising antitumor agents. Increasing number of researches reported the excellent performance of artemisinin and its derivatives in fighting against various kinds of cancer cell lines, and their clinical application prospect was shown to be broad. Some good reviews have been published [26–30]. In present review, we summarize some of the key issues in the development of artemisinin and its derivatives as antitumor agents evidenced by over 150 papers on this topic published in the last 5 years. By taking the activities, mechanisms, benefits, and limitations of antitumor-related artemisinins into consideration, we provide a significant sight for the current and future development in this promising field of cancer drug discovery.

## Antitumor Activity of Artemisinin

In the last 5 years, numerous new studies have shown that artemisinin and its derivatives can selectively kill various cancer cells, including leukemia [31–33], brain glioma [34], liver cancer [35, 36], gastric cancer [37, 38], breast cancer [11, 39–41], lung cancer [42–44], colon cancer [45, 46], B cell lymphoma [15, 47, 48], cervical cancer [49, 50], head and neck carcinoma [51], gall bladder cancer [52], nasopharyngeal cancer [53], osteosarcoma [54], esophageal cancer cells [55], rhabdomyosarcoma [56], schwannoma cells [57], pancreatic cancer [58], ovarian cancer [21], melanoma [59] and prostate carcinoma [16, 60]. Moreover, artemisinins have no cross resistance with traditional therapeutic drugs, and they can reverse the multi-drug resistance of tumor cells [61]. In terms of the pharmacokinetic properties, artemisinin-related compounds have the following features: fast-absorption, wide-distribution and quick-excretion. Therefore, the research of antitumor activities of artemisinin and its derivatives might represent a promising start to open a new avenue for cancer treatment.

### Antitumor Activity of Artemisinin on Leukemia

In the early 1990s, Chinese researchers firstly reported that artemisinin has inhibitory activity to the peripheral blood leucocyte 3H-TdR of leukemia patients, in a concentration-dependent manner. Since then, increasing number of literatures proved the anti-leukemia potentials of artemisinins, detailed in Table 1.

**Table 1:** Anti-leukemia cells activities of artemisinins

Compounds	Effect	Event/mechanism	References
Derivatives	Reversed multidrug resistance	Overcoming cross resistance	[31, 61]
DHA	Induced autophagy Inhibited tumor growth	Expression of LC3-II, caspase-3 activation, Down-regulation of TfR expression, and cell growth arrested in the G2/M phase	[62]
DHA	Induced apoptosis	Up-regulated the transcription factor FOXO3a	[63]
ART-838	Inhibited cell growth Reduced cell proliferation and clonogenicity Induced apoptosis Increased intracellular levels of ROS	Increased intracellular ROS levels	[64]
ART, AS, DHA	Induced apoptosis Increased ROS generation	Induced cytotoxicity and apoptosis by activated caspase 3/7 Induced ROS generation and lysosomal disruption	[65]
DHA	Inhibited tumor growth	Suppressed the expression of Bcr/Abl protein, Reduced the Bcr/Abl tyrosine activity of AKT and ERK1/2, suppressed NF-κB protein expression, Promoted the cytochrome *c* release and activated caspase3/9	[66]
DHA	Induced cell death	Inhibited the Bcr/Abl fusion gene at the mRNA level Inhibited the expression of Bcr/Abl and suppressed the activity of tyrosine kinase, suppressed the downstream signals of Bcr/Abl, reduced the tyrosine kinase activity of AKT and ERK1/2, promoted the cytochrome *c* release and activated caspase9/3	[67]
AS	Suppressed tumor growth Induced apoptosis	Suppressed the phosphorylation of p38, ERK, CREB, Chk-2, STAT5, and RSK proteins, Activated caspase-3, inhibited p38, ERK, STAT5, and CREB activation	[68]

As illustrated in Table 1, ART, DHA and AS got more attention in the treatment of leukemia during the recent 5 years [69–73]. To evade the existing problem of them, some novel artemisinin-derived compounds have been synthesized. These new compounds, including artesunic acid homodimers [32], 1,2,4-trioxane-ferrocene hybrids [31] and artemisinin-derived dimers and trimmers [74], have the ability to overcome multidrug resistance. After the combinations of artemisinin and some cholic acid derivatives, researchers found that the hybrids showed promising performance both in fighting against sensitive human leukemia CCRF-CEM cells and multidrug-resistant human leukemia CEM/ADR5000 cells. The IC_50_ values of the compounds are in the range of 0.019–0.192 μM against CCRF-CEM cells and between 0.345 and 7.159 μM against CEM/ADR5000 cells, respectively [75]. Additionally, amide hybrid was proved to be the most potent compound, which may help us to design new artemisinin-based hybrids by using amino bond as one part of the linker. Another modification strategy of artemisinins is to develop dimer derivatives of them. By using substituted chalcones as a linker, Rashmi Gaur et al. synthesized a series of artemisinin-derived dimers with considerable anti-leukemia activity [76].

### Antitumor Activity of Artemisinin on Breast Cancer

Recent researches of antitumor activity of artemisinins on breast cancer mainly focus on the nanoliposomal artemisinins [11, 39, 77–79]. Using a nanoparticle carrier to deliver drugs is a good way to improve the bioavailability and pharmacokinetic properties of the drugs. Among the multitude of nanoparticle systems, liposomal nanoparticles represent a class of better developed delivery vehicles [77]. Cumulative studies revealed that nanoliposomal formulation of artemisinins substantially increased the antitumor effects of artemisinin-related compounds. Despite of nanoliposomal artemisinins, researches also focus on the development of novel artemisinin-based compounds with anti-breast cancer properties [80]. A hybrid compound tethering DHA with diaryl-pyrazoline through ether linkage was found to exhibit potent activity in three breast cancer cell lines including MCF-7/Adr (GI_50_ = 18 nM), MCF-7 (GI_50_ = 210 nM) and MDA-MB-231 (GI_50_ = 210 nM) [81]. The inhibitory activity against MCF-7/Adr cells of the compound was proved to be 184-folds higher than DHA, indicating that the compound could developed to be a useful candidate for the treatment of drug-resistant breast cancer. During the exploration of the targets, phospho-translationally controlled tumor protein (phosphor-TCTP) was suggested to be one of the promising therapeutic target of DHA for advanced breast cancer [82] (Table 2).

**Table 2:** Anti-breast cancer cells activities of artemisinins

Compounds	Effect	Event/mechanism	References
Artemisinin dimers	Inhibited growth Induced cell death	Down-regulated the anti-apoptotic protein, survivin, and cyclin D1 Down-regulated the oncogenic protein HER2, HER, declined the wild type epidermal growth factor receptor (EGFR or HER1)	[77, 78]
DHA	Induced apoptosis Decreased cell proliferation	Caused nuclear phospho-TCTP overexpression Increased the Ki-67 expression, synergized with Doxorubicin and Trastuzumab	[82]
ART, AS	Arrested cell cycle Decreased cell proliferation	Increased miR-34a expression, down-regulated the expression of the miR-34a target gene, CDK4, miR-34a required functional p53	[83]
DHA	Induced apoptosis Induced G0/G1 arrest	Increased the expression of caspase-8, cleaved caspase-9, activated Bid, induced the release of cytochrome *c* from mitochondria into the cytosol, increased the expression of t Bim, decreased the expression of Bcl-2	[84]
Tehranolide	Inhibited proliferation Induced G0/G1 arrest Induced apoptosis Increased ROS levels	Modulated the PI3 K/AKT signaling pathway, increased cytochrome *c* and Bax, decreased BCL-2, down-regulated ayclin D1, released p27kip1	[85]
ART	Inhibited proliferation Induced G1 cell cycle arrest Induced growth arrest of tumorigenic	Down-regulated the transcript and protein levels of the CDK2 and CDK4 cyclin-dependent kinases, cyclin E, cyclin D1, and the E2F1 transcription factor	[86]

### Antitumor Activity of Artemisinin on Lung Cancer

Lung cancer has the characteristics of high incidence, considerable mortality, and poor prognosis. Moreover, its five-year survival rate remained at 15% because of the metastasis of the tumor cells. Clinical trials together with in vivo and in vitro experiments elucidated that artemisinin can inhibit and kill lung cancer cells [87–90] (Table 3).

**Table 3:** Anti-lung cancer cells activities of artemisinins

Compounds	Effect	Event/mechanism	References
AS	Enhanced radiosensitivity Induced cell cycle arrest	Increased the NO levels, down-regulated cyclin B1 mRNA expression	[42]
AS	Inhibited invasion Inhibited metastasis	Inhibited urokinase-type plasminogen activator (u-PA) activity, -protein and -mRNA expression, down-regulated MMP-2-, MMP-7- and u-PA-promoter/-enhancer activity, suppressed AP-1- and NF-κB-transactivation	[87]
AS	Influenced ribosomal activity, drug transport, cellular antioxidant defense Induced apoptosis Inhibited proliferation Arrested cell cycle	Related to tumor necrosis factor (TNF) and the tumor suppressor p53, influenced the activity of transcription factors regulating downstream genes, influenced by c-Myc and Max-mediated transcriptional control of gene expression	[88]
ART, DHA, AS	Inhibit proliferation Arrested cell cycle Decreased tumor growth Suppressed migration, invasion, cancer stem cells and epithelial-mesenchymal transition	Decreased the protein level of Wnt5-α/β, increased the levels of NKD2 and Axin2, down-regulated β-catenin	[91]
DHA	Suppressed proliferation Induced apoptosis Inhibited angiogenesis	Influenced the expression of VEGF receptor KDR/flk-1, enhanced the effects of chemotherapeutics	[92]
DHA	Induced apoptosis	Increased the concentration of Ca^2+^, activated p38	[93]

Studies demonstrated that the IC_50_ values of artemisinin and artesunate were 769.60 and 153.54 μM on A549 cell line [90]. AS is more potent than ART. The exploration of underlying mechanism of artemisinin and its derivatives in fighting against lung cancer suggested that Wnt/β-catenin represented a novel target for ART, DHA and AS in cancer treatment [91]. Ruling Shen et al. reported that combination of onconase (Onc) and DHA can synergistically suppress the growth and angiogenesis of non–small-cell lung carcinoma (NSCLC) both in vivo and in vitro. Importantly, there is no obvious adverse effects observed after the combined treatment of onconase and DHA [94].

### Antitumor Activity of Artemisinin on Liver Cancer

Inducing apoptosis and cell cycle arrest were thought to be two of the main antitumor mechanisms of artemisinin and its derivatives (Table 4).

**Table 4:** Anti-liver cancer cells activities of artemisinins

Compounds	Effect	Event/mechanism	References
DHA	Induced cell cycle arrest Induced apoptosis	Induced p21, inhibited cyclin B and CDC25C, depolarized mitochondrial membrane, released cytochrome *c*, activated caspase 9 and caspase 3, decreased Mcl-1 expression and increased the levels of Noxa and active Bak	[62]
DHA	Induced apoptosis	Reduced mitochondria membrane potential, released cytochrome c into cytoplasm, increased p53 and Bak, decreased Mcl-1, Ki-67 and p-ERK, activated caspase 3 and PARP	[95]
ART	Inhibited tumor cell invasion and metastasis	Down-regulated the protein levels of MMP2, activated Cdc42 by activating E-cadherin activity, improved cell–cell adhersion	[96]
ART, AS, ATM, DHA	Cytotoxic activity	Increased the expression of MDR1, MRP2 and MRP3	[97]

Combination use of histone deacetylase inhibitors (HDACi) and DHA has shown a significantly antineoplastic effect both in vivo and in vitro [95]. After the combined treatment with DHA and HDACi, the mitochondria membrane potential, the expressions of Mcl-1, p-ERK, Ki61, activated caspase 3, and PARP were decreased, while p53 and Bak expressions were increased. Therefore, DHA-induced apoptosis has been enhanced by the combination of HDACi. Furthermore, combination treatment of DHA and farnesylthiosalicylic acid or the glutaminase-1 inhibitor 968 also can enhance the antitumor efficacy of DHA in hepatocellular carcinoma cells [98, 99].

### Antitumor Activity of Artemisinin on Brain Glioma

Brain glioma is the most lethal cancer with extremely poor prognosis and high penetrability. Artemisinin and its derivatives have been shown to have considerable effects to fight against brain glioma (Table 5).

**Table 5:** Anti-brain glioma cells activities of artemisinins

Compounds	Effect	Event/mechanism	References
DHA	Inhibited proliferation	Induced autophagy	[34]
AS	Induce oxidative DNA damage	Resulted in DNA double-strand breaks (DSB) with phosphorylation of ATM, ATR, Chk1, and Chk2	[100]
DHA	Inhibited proliferation Induced apoptosis Induced cell cycle arrest	Increased Cleaved Caspase-3, decreased the expression of p-AKT, down-regulated AKT phosphorylation followed by Caspase-3 activation	[101]
ATM	Inhibited the migration and invasion Promoted the apoptosis	Inhibited the expression of MMP-2/9 and p-AKT	[102]
DHA	Inhibited proliferation Inhibited migration and invasion	Suppressed the expressions of a disintegrin and metalloproteinase 17 (ADAM17), and phosphorylated epidermal growth factor receptor and AKT (p-EGFR and p-AKT, respectively)	[103]

So far, combination treatment of DHA and temozolomide is the main strategy for using artemisinins as anti-glioma agents. After combined chemotherapy, autophagy molecular markers (Beclin-1 and LC3-B) expression was increased, while the expression of caspase-3 has not been detected with significant alteration. The results suggested that DHA can increase the tumor inhibition efficacy of temozolomide via inducing autophagy [34]. AS also enhanced the anti-proliferative effect of temozolomide on U87MG and A172 glioblastoma cell lines [23, 104]. Functional targeting paclitaxel plus artemether liposomes recently have been reported as a promising strategy for treating invasive brain glioma [105]. Combination treatment of artemether and shRNA-VCAM-1 not only significantly suppressed the migration, invasion and expression of MMP-2/9 and p-AKT, but also promoted the apoptosis of human glioma U87 cells [102].

### Antitumor Activity of Artemisinin on Colorectal Cancer

Colorectal cancer is the second most common malignant tumor and the fourth leading cause of cancer-related deaths worldwide. Recent studies have demonstrated that artemisnin-related compounds represent one kind of potential therapeutic strategy of colorectal cancer (Table 6).

**Table 6:** Anti-colorectal cancer cells activities of artemisinins

Compounds	Effect	Event/mechanism	References
DHA	Induced apoptosis Induced cell cycle arrest Accumulated ROS	Decreased the mitochondrial membrane potential; activated the caspase-3, caspase-8, and caspase-9; and increased the ratio of Bax/Bcl-2, activated the translation of apoptotic inducing factor (AIF) and the release of cytochrome *c* from the mitochondria	[106]
DHA	Induced growth inhibition Induced cell cycle arrest Induced apoptosis Inhibited migration	Decreased the expressions of NF-κB target gene products, such as PCNA, cyclin D1, and CDK4; and increased the expression of p21, cleaved-caspase-3, and cleaved-PARP	[107]
AS	Induced apoptosis	Reduced expression of Ki67 and increased CD31 expression	[108]

DHA treatment can significantly reduce human colorectal cancer HCT-116 cell viability in a concentration- and time-dependent manner. DHA triggered apoptosis in HCT-116 cells via the ROS-mediated mitochondria-dependent pathway [106]. In vivo experiments also showed that DHA have remarkable antitumor activity on colorectal cancer [107]. Synthesis and evaluation of novel artemisinin derivatives have found that some new artemisinin-related compounds also exhibited anti-colorectal cancer effects [109].

A clinical trial aimed to explore the anti-colorectal cancer effect of AS has shown that AS has anti-proliferative properties in colorectal cancer cells and is generally well tolerated. AS exposures led to 89% Ki67 reduction and 79% CD31 overexpression, respectively [108].

### Antitumor Activity of Artemisinin on Gastric Cancer

Artemisinin and its derivatives can inhibit the growth of gastric cancer both in vitro and in vivo. ART, AS, DHA and ATM are the most important artemisnin-related compounds which have been involved in the anti-gastric cancer cell activity exploration (Table 7). Researchers suggested that artemisnin-related compounds could serve as one kind of promising anti-gastric cancer cell agents or additional chemotherapeutic agents for treatment of gastric cancer.

**Table 7:** Anti-gastric cancer cells activities of artemisinins

Compounds	Effect	Event/mechanism	References
ART	Inhibited proliferation	Up-regulated p53, induce p27^kip1^ andp21^kip1^	[20]
AS	Inhibited proliferation Induced apoptosis	Reduced COX-2 expression, increased the expression of Bax and suppressed the expression of Bcl-2, activated caspase-3 and caspase-9, induced loss of mitochondrial membrane potential	[38]
AS	Inhibited cell growth	Induced calcium overload, down-regulated VEGF expression, up-regulated calpain-2 expression, produced a dose-dependent tumor regression	[38]
DHA	Inhibited proliferation Suppressed colony forming abilities Induced cellular senescence Induced cell cycle arrest Hindered the migration and invasion Induced apoptosis	Suppressed the expressions of PCNA, cyclin E, and cyclin D1, and up-regulated p21 and p27, down-regulated MMP-9 and MMP-2, suppressed Bcl-2, activated caspase-9 and PARP, increased miR-15b and miR-16 expression	[110]
DHA	Induced apoptosis	Up-regulated Bax, cleaved caspase-3 and -9 expressions, degraded form of PARP, downregulated the Bcl-2 expression and Bcl-2/Bax ratio, increased the phosphorylation of ERK1/2, JNK1/2 and p38 MAPK	[111]
ATM	Cytotoxic genotoxic effects	Induced both apoptosis and necrosis	[112]

Studies showed that DHA can strikingly inhibit the proliferation and colony-forming on three gastric cancer cell lines (SGC-7901, BGC823, and MGC803) by suppressing the expressions of proliferation markers (PCNA, cyclin E, and cyclin D1), and up-regulating the expression of p21 and p27 [110, 111]. Artesunate has been shown to have concentration-dependent anti-gastric cancer cell activity in vitro and in vivo through the mechanisms of inducing calcium overload, up-regulating calpain-2 expression, and down-regulating VEGF expression [113]. Furthermore, AS exhibited anti-proliferative effects and apoptosis-inducing activities on human gastric cancer cells in a dose-dependent manner. Overexpression of proapoptotic factor Bax, suppression of antiapoptotic factor Bcl-2 expression, activation of caspase-3 and caspase-9 and the loss of mitochondrial membrane potential were thought to be the potential mechanisms of AS anti-gastric cancer effects [38].

## Antitumor Mechanism of Artemisinins

As mentioned above, artemisinin and its derivatives have a variety of effects against cancer both in vitro and in vivo. Table 8 summarizes the main signaling pathways of artemisinin and its derivatives in cancer control.

**Table 8:** The cell process and signal pathway regulated by artemisinins

Signal pathway	Related protein (expression or activity)
Proliferation pathway	Suppressed the transcription and protein expressions of the CDK2 and CDK4 cyclin-dependent kinases, cyclin E, cyclin D1, and the E2F1 transcription factor, up-regulated miR-34a expression correlating with down-regulation of the miR-34a target gene (CDK4), decreased Wnt5-α/β expression, increased the expressions of NKD2 and Axin2, down-regulated β-catenin, suppressed the phosphorylation of ERK1/2, inhibited the mRNA and protein expression of ERK1/2, suppressed the transcription and expression of ERK1/2 downstream effectors c-Fos and c-Myc, up-regulated expression of p53, inhibited calmodulin (CaM) and phosphodiesterase (PDE1), accumulated Camp, activated cAMP-dependent protein kinase A (PKA)
Apoptotic pathway	Decreased p53 expression [50]; up-regulated Bax expression, down-regulated Bcl-2, Bcl-xL and Procaspase-3, increased caspase-9 activation [55]; induced the translocation of apoptotic inducing factor (AIF) and the release of cytochrome *c* from the mitochondria, decreased the mitochondrial membrane potential, activated the caspase-3, caspase-8, and caspase-9 [106]; and increased the ratio of Bax/Bcl-2; increased ROS level, triggered the intrinsic pathway of apoptosis [114], activated p38 MAPK [115]; increased miR-16 expression and decreased COX-2 expression [38] and PGE2 production [19]; decreased Mcl-1 expression, increased Bak and Noxa expression [62]; inhibited ERK phosphorylation [95]; induced necroptosis [57]; decreased HSP70 expression [116]
Cell cycle	Reduced the transcription activity of CDK2, CDK4, cyclin E [55]; increased the expression of CD71 [117]; up-regulated the expression of p21^Cip1^ and p27^Kip1^ [118]; decreased G2/M-associated proteins cyclin B1, CDC2 and MDM2 expressions, suppressed the expression of Forkhead box protein M1 (FOXM1) [51]; increased miR-34a expression, down-regulated miR-34a target gene [83]
Invasion and metastasis	Down-regulated MMP-9 and MMP-2 [110]; suppressed Rac1 siRNA deactivates NF-κB activity [107]; down-regulated the expression of ADAM17, p-EGFR and p-AKT [103]; inhibited the transcription of u-PA, MMP-2 and MMP-7, suppressed AP-1 and NF-κB-transactivation [87]; activated Wnt/β-catenin signaling pathway [91]; reduced vWF expression and macrophage infiltration, decreased expression of pFAK [21]
Angiogenesis	Reduced expression of the vascularization-related proteins HIF-1α and VEGF [119]; reduced the level of KDR, decline the transcriptional activity of ανβ3, reduced the levels of MMP2, MMP9 and BMP1
Oxidative damage reaction	Reduced the level of CD71 [120]; increased intracellular levels of reactive oxygen species (ROS) [64, 115]
Chemosensitivity and radiosensitivity	Down-regulated RAD51 and impair DNA DSBs repair, induced DSBs and inhibited the clongenic formation of tumor cells [121]; activated the MAPK pathway by TGF β often in a DPC-4independent manner, down-regulated P15/CDKN1B, up-regulated CDCA4 and CDC2L6 with DPC4 activation [122]; increased Cyclin B1 expression, affected multiple pathways including RNA transport, the spliceosome, RNA degradation, p53 signaling and MAPK [123]; elevated amount of cH2AX foci/nucleus, attenuation of survivin expression [23]; increased the NO level [42]
Microenviroment	Reduced the number of Treg [124], increased the proportion of IFN-γ/I-4, reduced PGE2, inhibited the produce of NO and cytokines (IL-1-b, IL-6, TNF-α, VEGF) [125]

### Inhibiting the Proliferation of Tumor Cells

In normal cells, cell growth and division are controlled by the mutual interaction of cell cycle protein cyclin, cyclin-dependent kinase (CDK) and cyclin-dependent kinase inhibitor (CKI) [52]. However, tumor cells have strong proliferative ability due to the amplification of growth signal, disabled regulation of testing point and cell mutations. Artemisinin and its derivatives can arrest cell cycle in the tumor cells, mainly by interfering cell cycle kinetics or blocking the proliferation related signaling pathway [16].

So far, the main mechanism of artemisinin and its derivatives to inhibit tumor cell proliferation was thought to be closely associated with the selective inhibitory effect of the transcriptional activity and protein expression levels of CDK2/4 cyclin-dependent kinases, cyclin E, cyclin D1, and the E2F1 transcription factor. Therefore, artemisinin and its derivatives can inhibit the activity of related catalytic enzyme protein in tumor cell proliferation, and subsequently, inhibit the proliferation of tumor cells [86]. Both ART and AS can increase the expression of miR-34a in a dose-dependent manner accompanied with the suppression of the miR-34a target gene, CDK4. The findings verified that miR-34a is a pivotal factor for the anti-proliferative activity of ART and AS [83].

The anti-proliferation activity has also been proved to be closely related to cell cycle arrest, which is partially dependent on Wnt/β-catenin inactivation [91]. ART, DHA, and AS remarkably inhibited the proliferation of human NSCLC A549 and H1299 cells by arresting cell cycle in G1 phase. Additionally, ART can inhibit proliferation in rat pituitary adenoma GH3 cells by caspase-dependent pathways [24]. DHA can inhibit the proliferation of human umbilical vein endothelial cells (HUVECs) by reducing the phosphorylation of ERK1/2, down-regulating the mRNA and protein expression of ERK1/2 and inhibiting the transcription and protein expression of ERK1/2 downstream effectors c-Fos and c-Myc [126]. AS exhibited significant anti-proliferative effects on naive CD4^+^ T cells by reducing the expression of cell surface protein CD25 (IL-2 receptorα chain) and CD69 [127]. Studies also showed that ART can synergistically interact with other antitumor agents. For instance, 7P3A, which consists of 70% 25-methoxyl- dammarane-3β, 12β, 20-triol and 30% artemisinin inhibited MDA-MB-231 cell proliferation and induced cell cycle arrest through down-regulating the expression of testes-specific protease 50 (TSP50) [128].

### Inducing Tumor Cells Apoptosis

Apoptosis plays a vital role in tumor cure. Loss or inhibition of apoptosis process is likely to be the trigger for cancer occurrence and drug resistance of tumor cells. Mitochondrial pathway is one of the most essential way in cell apoptosis process. Artemisinin reacts with ROS of tumor cells, leading to the increase of ROS in tumor cells [114], qualitative oxidation of mitochondrial membrane, decrease of permeability, and mitochondrial membranous potential. Therefore, cytochrome *c* and AIF subsequently released into the cytosol. Caspase-8/9 gets activated, and finally activated caspase-3 induces cell apoptosis [106]. Artesunate induced cell apoptosis in a dose-dependent manner by up-regulating the ROS levels and activating p39 MAPK [115]. Artemisinin-induced pro-apoptotic effect was also verified to be p53-dependent [50]. Additionally, dihydroartemisinin can increase Bax expression and caspase-9 activation. It could suppress Bcl-2, Bcl-xL and procaspase-3 as well, thereby, accelerating DHA-induced apoptosis of esophageal cancer cells [55]. Moreover, DHA-induced apoptosis has been proved to be implicated to the mitochondrial membrane depolarization, release of cytochrome *c*, Bcl-2 family proteins expression and DNA fragmentation [62, 95]. COX-2 down-regulation [19, 38], HSP70 inhibition [116], and necroptosis [57] also play significant roles in artemisinins-induced apoptosis.

### Cell Cycle Arresting

Tumor cells have the property to disorder G1 to S phase differentiation process to increase the number of cells which can enter S phase. DHA can arrest tumor cells in G0/G1 phase to block the growth of tumor cells.

DHA exposure resulted in downregulation of cell cycle-related proteins, such as cyclin E, CDK2 and CDK4. Cyclin E, CDK2, and CDK4 are important complexes responsible for the progression of cells through the G1 phase of cell cycle and initiation of DNA replication [55]. Artemisinin markedly arrested retinoblastoma cells at G0/G1 and S phase but no effect on the G2/M phase through increasing the expression of CD71. More importantly, ART even can arrest cell cycle of multidrug-resistant retinoblastoma cells [117]. Combinational therapy of Halofuginone (HF) and Artemisinin synergistically induced G1/G0 arrest of HCT116 cells and MCF-7 cells. Up-regulation of p21^Cip1^ and p27^Kip1^ are responsible of this synergistic anticancer effect [118].

### Inhibition of Tumor Cell Invasion and Metastasis

The most essential effect of artemisinins is to inhibit the tumor cell invasion and metastasis in aggressive solid tumors. Numerous researches have proved that artemisinin and its derivatives can inhibit the invasion and metastasis of tumor cell without bias of cell line. For instance, AS can considerably inhibit invasion and metastasis in NSCLC. The main mechanisms are partly due to the down-regulation of MMP-2 and MMP-7 mRNA/protein, the inhibition of urokinase-type plasminogen activator (u-PA) activity, -protein and -mRNA expression and the suppression of u-PA-promoter/-enhancer activity, AP-1-and NF-κB-transactivation [87]. Similar result was reported by Han et al. They also found that DHA manifested remarkably anti-metastatic effect through inhibiting NF-κB activity [107]. DHA is also able to reduce glioma cell invasion and metastasis via inhibiting ADAM17 mRNA and protein expression and decreasing EGFR and AKT phosphorylation [103]. The down-regulation of MMP-2 and MMP-9 has been verified to be contributed to the anti-invasive and anti-metastatic effects of DHA [110]. A recent study has shown that ART, DHA and AS can inhibit invasion and migration of A549 and H1299 cells via depressed Wnt/β-catenin signaling pathway [91]. Moreover, in vivo experiments illustrated that DHA can significantly inhibit migration and invasion of ovarian cancer cell by reducing vWF expression and macrophage infiltration [21].

### Antiangiogenic Effects

Tumor angiogenesis occurs mainly by boosting the vascular permeability, promoting basal stem cell migration, division and proliferation, accelerating the formation of the vessel lumen, and ultimately, promoting the growth of tumor blood vessels through the VEGF receptor secreted by tumor cells. Thus, obstacle of any one mechanism of the growth of tumor cells and endothelial cells, would be able to inhibit tumor cell growth, and subsequently suppress tumor proliferation. Artemisinin and its derivatives can reduce the expression of VEGF and vascular endothelial cell receptor in tumor cells, so that the new angiogenesis can be blocked, and proliferation and metastasis of tumor cells can be inhibited [94]. Moreover, combinational treatment of DHA and onconase can enhance the suppression of endothelial cell tube formation. DHA coupled with cisplatin (CDDP) has been shown to exhibit anti-angiogenic effect by suppressing expression of the vascularization-related proteins HIF-1α and VEGF both in vivo and in vitro [119]. AS combined with captopril also exhibited synergistic effects on inhibiting new vessel formation and growth [129].

### Oxidative Damage Reactions

Tumor cells require more iron to maintain their rapid proliferation. A consensus opinion is that artemisinin-related compounds exert their antitumor activities through oxidative damage reactions. DHA exhibited ROS-independent antitumor mechanism by down-regulating cell-surface TfR1 level through an unexpected endocytic pathway, leading to the decline of TfR1 mediated iron uptake and deficiency of cellular iron stores [120]. DHA, AS, Artemisinin-derived dimer ART-838, and novel natural sesquiterpene lactone with an endoperoxide group (tehranolide) have been shown to be able to inhibit the growth of tumor cells by increasing intracellular levels of ROS [64, 115, 130, 131].

### Enhance the Sensitivity of Chemotherapy and Radiosensitivity

During the treatment of cancer, chemotherapeutic drugs sometimes can stimulate the normal cells to produce chemicals which might accelerate the growth of tumor cells. Thus, tumor cells can gradually become resistant to chemotherapy drugs. Somatic mutations or homozygous deletions of DPC4/SMAD4, TP53 and P16/CDKN2A genes have been elucidated to have correlation with in vitro drug responses. The activation of MAPK pathways by TGFβ is often in a DPC-4-independent manner. DPC4 activation also resulted in the down-regulation of P15/CDKN1B and up-regulation of CDCA4 and CDC2L6 [122]. Artesunate has been indicated to be able to compromise the repair of DNA DSBs in ovarian cancer cells through down-regulating the formation of RAD51 foci and homologous recombination repair (HRR). Thus, RAD51 has been identified as a key component for developing AS as a sensitizing agent in chemotherapy [121]. In addition, AS can also increase radio sensitivity of tumor cells, though the mechanism was complicated as more than 200 genes were involved. AS exposures remarkably influenced multiple pathways including RNA transport, the spliceosome, RNA degradation, p53 signaling and MAPK [123]. Moreover, AS elicited radio-sensitizing effect by down-regulating apoptosis protein surviving [23]. Some researches illustrated that NO production is associated with the radiosensitivity. AS exhibited potential ability to enhance radiosensitivity of human NSCLC A549 cells by increasing the NO level within irradiated A549 cells [42].

### Regulating Tumor Microenvironment

In tumor microenvironment, tumor associated macrophage (TAM) is thought to have multiple functions of promoting tumor growth and metastasis, such as secreting growth factors and protease, promoting angiogenesis, and inhibiting adaptive immunity. DHA can inhibit macrophage infiltrate into the tumor in situ and metastases, indicating a new mechanism of the anti-cancer activity of artemisinin-related compounds [21].

Regulatory T cells (Treg) is a pivotal member of tumor microenvironment, which can lead to the silence of immune response. The accumulation of Treg cells in early tumor tissues is associated with tumor development and the poor prognosis. In mouse xenograft models of breast cancer, artemisinin remarkably reduced the number of Treg in tumor matrix, and increased the proportion of IFN-γ/I-4 in the supernatant of spleen cells [124], leading to the inhibition of the immune microenvironment on tumor development. Artemether can enhance delayed-type hypersensitivity and antibody of coagulation in normal mice, reduce the CD4^+^CD25^+^ Foxp3^+^ Treg cells in tumor-burdened mice spleen, increase the produce of IL-4 and IFN-γ [132]. Similar results were found by Noori and Hassan [133]. During their research they have also detected the decrease of CD4^+^, CD25^+^, Foxp3^+^, Treg cells, promotion of IFN-γ and inhibition of IL-4 secretion which can trigger the Th2 switch to Th1 pathways. DHA can reduce PGE2, inhibit the produce of NO and cytokines (IL-1-b, IL-6, TNF-α, VEGF) to regulate immune functions [125]. These researches documented that regulating tumor microenvironment is one of molecular mechanism of antitumor activity of artemisinin-related compounds.

## Clinical Trials of Artemisinins as Anticancer Drugs

Hosoya K and co-workers from The Ohio State University evaluated the clinical toxicity and activity of artemisinin in dogs with spontaneous tumors through oral administration [134]. In their study, 24 dogs were separated into two groups randomly, and each group received low-continuous dose (3 mg/kg q 24 h) or high-dose intermittent (three doses of 45 mg/kg q 6 h repeated q 1 week) of artemisinin per os for 21 days. However, only 11% of the low-dose group and 29% of the high-dose group were observed with anorexia. Both groups were well tolerated with artemisinin in dogs through oral administration. Unfortunately, both groups exhibited low bioavailability, thus, parenteral administration should be taken into consideration for further studies. Additionally, it is obliged to develop new artemisinin-related compounds with good bioavailability and desirable antitumor property.

In general, the clinical trials of artemisinins are mainly concentrated on artesunate. A group from the University of Gothenburg first characterized the population pharmacokinetics of AS and DHA in patients with metastatic breast cancer and the relationship between salivary and plasma concentrations of DHA during long-term (> 3 weeks) daily oral AS administration [135]. Their study elucidated that saliva sampling could be used in pharmacokinetic investigation to offer a cheaper sampling alternative as drug concentrations in saliva are in equilibrium with that in arterial blood. Furthermore, the metabolism of AS was suggested to be autoinduction. After long-term daily oral AS, a 24.9% increase in apparent elimination clearance of DHA was observed. In 2015, Sanjeev Krishna and co-workers also reported their clinical research about the antitumor property and tolerability of oral AS in colorectal cancer [108]. 67% of patients in AS group (n = 12) and 55% of patients in placebo group (n = 11) were developed > 7% cell apoptosis. Ki67, which is thought to be a significant biomarker of prognosis in colorectal cancer, was found to be decreased after the 200 mg daily oral treatment of AS, while the expression of the CD31 was increased. Bayesian analysis showed that the probabilities of AS treatment effect reducing Ki67 and increasing CD31 expression were 0.89 and 0.79, respectively. The results indicated that the antitumor effect of AS was also related to the immunohistochemical outcomes. During this study, only one patient treated with AS developed recurrent colorectal cancer, while six patients treated with placebo were observed with recurrent colorectal cancer. However, two adverse events possibly related to AS were observed, which urged us to do more comprehensive clinical studies with artemisinin-related compounds to complete the information of this kind for promising antitumor agents. In order to determine the maximum tolerated dose (MTD) and dose-limiting toxicities (DLTs) of intravenous AS, Deeken et al. performed a phase I study to explore the utility of AS to fight against solid organ malignancies [136]. 19 patients were enrolled in the study, and 18 of them were evaluable for toxicity, while 15 of them were evaluable for efficacy response. At the dosage of 25 mg/kg, both two patients experienced DLTs, while only one of six patients at dosage of 12 and 18 mg/kg were suffered from DLTs. Hence, the MTD was found to be 18 mg/kg using a day 1 and 8 on a 21-day cycle intravenous push administration. Different kinds of toxicities were observed, including anemia, nausea/vomiting fatigue and anorexia, which were reported previously with the use of AS in malaria treatment. Moreover, liver function and electrolyte disturbances also have been observed in a significant percent of patients. Therefore, pre-medication aimed to prevent nausea or hypersensitivity reactions should take into consideration in future application.

Some previous literatures showed that artemisinins might have adverse effects related to the auditory and vestibular system [137–139]. Considering these concerns, an audiological assessments of AS based on 4 weeks oral intake of AS in doses up to 200 mg daily on 23 female patients with metastatic or locally advanced breast cancer were performed [140]. Desirably, all the patients involved in the treatments were well tolerated considering neuro-audiological function. Only two patients were observed with ongoing subclinical hearing loss and one patient with ongoing tinnitus. The results remind us that comprehensive audiological assessments should be added into clinical trials, while investigating the antitumor effects of oral AS.

In fact, over the past two decades, the researches about antitumor activities of artemisinin-related compounds were increasingly reported. However, literatures about the clinical trials were rarely to be found, and very fewer patients were involved in the most of the available clinical trials. Therefore, in the further clinical trials, more patients should be added into the researches, and more comprehensive factors should be taken into consideration to promote the development of clinical application of the artemisinin-related compounds.

## New Directions

### Immune Regulation of Artemisnins

Tumor immunotherapy is becoming one of the main therapeutic strategies of the cancer treatment. Deeper investigations showed us that artemisinins also cause immune response to the cancer cells [7, 10, 40]. Five kinds of immune cells, including DC cells, CIK, natural killer cells (NK cells), natural killer T cells (NKT cells), and CD3AK cells construct the overall line of defense for cancer treatment. The NK cells are one kind of cells of innate immune system, which can kill the tumor stem cells by stimulating the lytic granule exocytosis to prevent the recurrent and metastasis of cancer [141]. Hence inducing exocytosis of lytic granules of NK cells is a promising strategy to develop targeted cancer therapy [142]. Youn Kyung Houh and co-workers first reported that artemisinin exhibited powerful antitumor effect by activating NK cells [143], which provide a new prospect for the exploration of the mechanism of antitumor action of artemisinins. The study demonstrated that ART can enhance the cytolytic activity of NK cells in a dose-dependent manner, while the stimulation of granule exocytosis was evidenced by the up-regulation of the expression of CD107ain NK cells. The phosphorylation of Vav-1, a downstream signaling molecule for NK activating receptors, was quickly up-regulated followed by the increasing of ERK 1/2 phosphorylation. The research pointed out that artemisinin can promote the degranulation of NK cells through the stimulation of signaling molecules of the NK activating receptor, thus, resulting in the potent antitumor activity of artemisinin.

With the development of immunology, tumor immunotherapy is about to claim a seat in the therapeutic pantheon of oncology, next to surgery, chemotherapy and radiation therapy [144]. Hence, further exploration of the roles of artemisinins in tumor immunotherapy might expand our understanding of artemisnins.

### The Activator of Artemisnin

It is commonly accepted that the endoperoxide bridge of the artemisnins is vital for its antitumor activity, while ferrous iron (either in free ferrous form or in haem form) was thought to be the main activator of the antitumor activity of artemisnins [145]. However, the exact activator is still in controversy.

Over years, free ferrous iron was proposed to be the principal activator of artemisinin [6, 8, 26, 29]. However, by presenting an unbiased chemical proteomics analysis, Wang et al. have pointed out that the activation of artemisnin is predominantly haem dependent [29, 146]. In order to identify the targets of artemisnin, an alkyne-tagged artemisinin analogue coupled with biotin (named as AP1) was designed and synthesized. During the study, they found 124 targets of artemisinin in parasite but also elucidated that artemisinin can be activated by haem directly. API alone cannot bind to the ornithine aminotransferase (OAT), which is involved in the key metabolic pathways of the parasite. API binding need the exposure of haemin and can be enhanced by L-ascorbic acid (Vc)) Na_2_S_2_O_4_ or glutathione (GSH) reagents which can reduce haemin to haem [147], while the addition of ferrous iron have no effects on API-OAT binding. A new artemisinin-based activity probe ART-TPP-Alk was then synthesized. 321 and 860 proteins which can covalently bind to artemisnin in HCT116 cells have been figured out by the use of AP1 and ART-TPP-Alk, respectively. The chemical proteomics and cell viability experiments demonstrated that free heme could effectively activate ART, while free ferrous iron has minimal effects on the activation of ART, and the modulation of endogenous heme could also affect anticancer activity of ART. The results validated that free heme is the key activator in cancer cells [148]. Based on the researches, the anticancer effects of artemisinin combined with a clinically used heme synthesis precursor amino levulinic acid (ALA) was evaluated. Desirably, the specific cytotoxicity of ART toward colorectal cancer (CRC) cells can be enhanced with the addition of ALA [149]. Their results encouraged us to take up the mechanism of actions starting with the activator, and find new artemisinin-related antitumor compound which can act with the activator.

### Nanoplatform Enhance the Effectiveness of Artemisnins

Although there are so many researches signified that artemisinin-related compounds have potent antitumor properties in virtue of their few adverse effects and greater tolerance by patients [150], the clinical application of artemisnins was hardly to be found. The main obstacle of artemisnins for clinical applications was poor aqueous solubility and low bioavailability [136], leading to the requirement of high dosage of artemisnins for greater anti-tumor effects. Unfortunately, with the increasing usage of artemisnins, the side effects of them usually became more remarkable [151]. Worse still, as one kind of radical-induced therapeutic drugs, it is difficult for artemisinins to achieve local treatment [152]. Hence, the urgent need is to find a potential method to address these problems. Nanoparticles, a key tool in targeted cancer therapy, can achieve a drug-targeted distribution and increase the bioavailability of the drug to improve the treatment efficacy [153].

In recent years, an increasing number of researches focus on the development of artemisinin-based nanoplatforms to promote the therapeutic efficiency of cancer treatment [11, 59, 79, 152, 154–156]. Zhang et al. recently synthesized two kinds of nanoplatforms to apply into artemisinin-based cancer treatment [157, 158]. One kind of nanoplatform that they developed was a visible-light-sensitive nanoplatform (HA-TiO2-IONPs/ART). TiO_2_ was grafted with Fe_3_O_4_ to form magnetic titania nanocomposites (TiO_2_-IONPs) to serve as visible-light sensitive photocatalysts [159]. Polyethylenimine (PEI) was grafted on TiO_2_-IONPs by Fe–N coordination bond. By linking hyaluronic to the nanomaterials (HA-TiO_2_-IONPs), the biocompatibility, dispersion stability and cytophagy ability of TiO_2_-IONPs have been improved. Artemisinin was at last loaded on the carrier to obtain the final delivery nanoplatform (HA-TiO_2_-IONPs/ART). As the main part of the nanoplatform, TiO_2_-IONPs can absorb visible light to generate ROS from tumor photodynamic therapy (PDT). Additionally, TiO_2_-IONPs can be degraded in acid environments to release ferrous iron. Since tumor site is a slightly acidic environment [160], TiO_2_-IONPs will release the ferrous iron when they reach the target tumor site. Thus, artemisinin and ferrous iron can be delivered into cancer cells simultaneously. In vitro and in vivo experiments were carried out to evaluate the antitumor effect of HA-TiO_2_-IONPs/ART. This ferrous iron and artemisinin co-delivery system finally proved to be the promising anti-tumor agents. Another nanoplatform they have developed was similar to HA-TiO2-IONPs/ART. Mesoporous Fe_3_O_4_, a kind of alternating magnetic field (AMF) and tumor-responsive material, was used to encapsulate artemisinin. Then, the outer surface of mFe_3_O_4_ was capped with HA (HA-mFe_3_O_4_/ART). HA-mFe_3_O_4_/ART can bring ART and ferrous iron into MCF-7 cells, and release Fe2^+^ and ART at the same site. Moreover, AMF irradiation could enhance antitumor activity of HA-mFe_3_O_4_/ART by converting electromagnetic wave into heat for tumor thermal therapy and promoting the generation of ROS for tumor PDT [157]. Similarly, based on the ROS-mediated cellular apoptosis mechanism, a novel artemisinin-based Fe^2+^-mediated ROS-generating nanodrug system with high ART loading capacity, acidic degradability, and biocompatibility have been discovered [3]. The smart nanodrug system can efficiently accumulated at the tumor site. Therefore, the weak acidic tumor microenvironment could accelerate the release of Fe^2+^ to produce ROS. In addition, a near-infrared light could increase the local temperature of tumor to accelerate the ROS generation. This localized ROS generation is a promising way to fight against malignant cells and solid tumor.

Lidong Liu and co-workers developed a different kind of artemisinin-loaded mesoporous NiO nanoplatform based on mesoporous NiO nanoparticles (mNiO) and terbium (Tb) complexes as vehicle [152]. The mNiO, which is a new pH-responsive material is stable at physiological pH 7.4. However, tumor cells always turned out to be acid. Thus, under tumor microenvironments, mNiO degrade to release Ni^2+^ which could lead to the cleavage of the endoperoxide bridge of ART and produce free radicals to kill tumor cells. It is noteworthy that mNiO can also be used as an photothermal conversion agent for cancer photothermal therapy (PTT) due to its remarkable near-infrared absorbance. The outstanding performance in both T2-weighted magnetic resonance imaging (r^2^ = 6.30 (mg mL^−1^)^−1^ s^−1^) and luminescence imaging suggested that this natural drugs-based nanoplatform can serve as a synergistic therapeutic strategy for cancer treatment. In vivo and in vitro experiments also confirmed the good performance of the agent. Other researches also demonstrated that artemisinin-based nanoparticle delivery systems could particularly enhance the antitumor efficiency of artemisinin-related compounds [161, 162]. Therefore, the development of artemisinin-based nanoplatform is an effective way to find potent artemisinin derivatives with good bioavailability and targeting property.

### Artemisinin-based Synthetic Hybrid Compounds

Combinational therapy is widely used in cancer treatment to overcome the drug resistance. Nevertheless, it is a challenge to choose the proper drugs and doses for the combinational therapy due to the difference in chemical and pharmacokinetic properties of the drugs. The drug–drug interactions which might exist are also the concerns that we should take into consideration. Additionally, combinational therapy always needs to speed more money, which will cause an additional burden for the patients [163]. Therefore, the hybridization of bioactive natural products became a promising approach to overcome the above problems and obtain new specific anticancer agents [74, 163, 164]. Two or more natural product fragments combine with each other leading to new structure possessing with better biological activities via covalent bonds [5, 31, 74, 165, 166].

Annemarie Ackermann and co-workers linked artesunate with betulinic acid (BETA) to generate a hybrid compound 212A with desirable anti-glioma activity [167]. Compared with its parent compounds AS and BETA, 212A was more efficient. Noteworthy, glioma cell migration can be suppressed by the hybrid 212A. However, BETA alone almost has no effects on glioma migration. Conjugates of DHA and some marketed chemotherapeutic agents, including chlorambucil, melphalan, flutamide, aminoglutethimide, and doxifluridine also have been synthesized to evaluate their antitumor activities of ovarian cancer cells [168]. Among all the conjugates, artemisinin and melphalan hybrid compound (AS4) exhibited the best potential in fighting against ovarian cancer with minor cytotoxicity to normal cells. The inhibitory activity of AS4 has more potency than DHA and melphalan, as the IC_50_ values of AS4 are 0.86 μM to A2780 cells and 0.83 μM to OVCAR3 cells, while the IC_50_ values of DHA are 4.75 μM to A2780 cells and 5.63 μM to OVCAR3 cells, and the IC_50_ values of melphalan are 23.18 μM to A2780 cells and 11.61 μM to OVCAR3 cells, respectively. AS4 can selectively induce the human ovarian cancer cell apoptosis, increasing the expression of caspase 3/7, and PARP, down-regulating the expression of Bcl-2. Reduced total AKT and dephosphorylation of AKT, mTOR and ERK indicated that AS4 could lead to the inactivation of the PI3 K/AKT and MAPK/ERK pathway in a dose-dependent manner. Furthermore, AS4 can inhibit cell-cycle progression at S-phase by down-regulating the expressions of CDKs and cyclins and up-regulating the expression of the CDK inhibitor p21 [168]. Nine new hybrid molecules of artemisinin and bile acid moieties were found to have the potentials to fight against drug-sensitive CCRF-CEM leukemia cells in a IC_50_ range of 0.019–0.192 μM and the multidrug-resistant leukemia sub-line CEM/ADR5000 in a IC_50_ range of 0.345–7.159 μM, respectively [31].

Above all, artemisinin-based synthetic hybrid compounds can enhance biological potential of artemisinins and improve the chemotherapeutic efficiency of the treatment. The hybrid approach opens up the possibility of combination of virtually any molecular structure and expands the exploring scope of chemotherapeutic agents [167].

## Future Development of Artemisinins as Anticancer Drugs

Tumor, a main disease which seriously threats human health, has always been a focus of medical field. In recent years, a lot of new chemotherapeutic drugs have been approved and radiotherapy technology is increasingly more mature. However, the sensitivity of tumor cells has decreased. Worse still, as most common chemotherapeutics still have many undesirable side effects, many patients even cannot continue the treatment. Therefore, the request for new drugs with high anticancer activity and low incidence of adverse effects remains a prevailing issue in clinical oncology.

As described above, artemisinin and its derivatives, the first-line drug of malaria, also have manifested remarkable effects on tumor treatment. Artemisinins can reverse chemotherapeutic resistance of tumor cells with low cytotoxicity in normal cells. Hence, tumor cells can become sensitive to chemotherapy drugs again, and the antitumor effect will be increased. Although numerous animal experiments and laboratory tests have been performed to explore the antitumor effect of artemisinins, our understanding of artemisnins is limited. In order to develop artemisinins as one kind of anticancer agents with high potency and low toxicity and make full use of artemisnins, further research should be done and we have a long way to go.

Here are some key issues that we need to explore: (1) The selective damage mechanism of artemisinin to cancer cells remains elusive, and the toxic mechanism of most artemisinin derivatives to normal cells still need further study. (2) It is difficult for artemisinin to achieve the antitumor goal of clinical therapy alone. Combination of artemisinin and its derivatives with traditional chemotherapeutic drugs can significantly enhance the anti-cancer effect of other chemotherapy drugs without any obvious adverse effects. Especially, in the cancer cells with chemotherapeutic drug resistance, artemisinin has shown significant antitumor activity, suggesting that artemisinin has the potential to be a combined chemotherapy drugs to reduce resistance of cancer cells with traditional chemotherapy drugs. However, proper combinations and doses is a tough issue to be resolved. (3) So far, most of the anti-cancer mechanism researches of artemisinins are mainly concentrated on artemisinin, dihydroartemisinin and other simple monomers. The researches on the anti-cancer mechanism of artemisinin derivatives and their polymers are still an important breakthrough point, and the structure–activity relationship of artemisinin derivatives antitumor activity still needs to be investigated. (4) Many studies have shown that different artemisinin compounds have different anti-cancer effects, indicating that the anticancer activity of artemisinins have selectivity. Artemisinins might cause antitumor activity in cancer cells through specific targets. So far, no exact target of antitumor-related artemisinins has been discovered. A recent research have found 860 proteins can bind with ART, this enables us to find more precise targets of artemisnins [148]. (5) Artemisinins not only have selective killing effect on tumor cells, but also have the ability to adjust the tumor microenvironment, anti-angiogenesis and inhibit the metastasis of tumor cells. Nevertheless, the related molecular mechanism has no systemic and clear conclusion. Therefore, in the future anticancer drugs research, the mechanism of artemisinin to kill tumor cells and to adjust microenvironment needs to be explored. The identification of the direct target of artemisinins and to establish a reasonable drug screening platform will provide an essential research foundation to the development of novel anti-cancer drugs which have clinical value.
